# Seroprevalence and risk factors of *Toxoplasma gondii* and *Neospora caninum* infection in black goats in Yunnan Province, Southwestern China

**DOI:** 10.3389/fvets.2022.975238

**Published:** 2022-10-11

**Authors:** Xiao-Hui Hu, Shi-Chen Xie, Qin-Li Liang, Li-Xiu Sun, Zhao Li, Jian-Fa Yang, Xing-Quan Zhu, Feng-Cai Zou, Jun-Jun He

**Affiliations:** ^1^Key Laboratory of Veterinary Public Health of Yunnan, College of Veterinary Medicine, Yunnan Agricultural University, Kunming, China; ^2^College of Veterinary Medicine, Shanxi Agricultural University, Taigu, China; ^3^State Key Laboratory of Veterinary Etiological Biology, Key Laboratory of Veterinary Parasitology of Gansu, Lanzhou Veterinary Research Institute, Chinese Academy of Agricultural Sciences, Lanzhou, China; ^4^Hunan Provincial Key Laboratory of Protein Engineering in Animal Vaccines, College of Veterinary Medicine, Hunan Agricultural University, Changsha, China; ^5^State Key Laboratory of Conservation and Utilization of Bio-Resources in Yunnan and Center for Life Science, School of Life Sciences, Yunnan University, Kunming, China

**Keywords:** black goats, Southwestern China, *Toxoplasma gondii*, *Neospora caninum*, seroprevalence, risk factors

## Abstract

*Toxoplasma gondii* and *Neospora caninum* are two obligate intracellular protozoan parasites that can cause reproductive failure and production losses. To date, there is no data of *T. gondii* and *N. caninum* seroprevalence in black goats in Yunnan Province, southwestern China. In the present study, a total of 734 serum samples were collected from black goats in four different counties of Yunnan Province. 734 and 590 serum samples were examined for antibodies against *T. gondii* and *N. caninum* by using MAT and indirect ELISA, respectively. A total of 123 and 76 samples were *T. gondii-*positive and *N. caninum*-positive, respectively. The overall seroprevalence of *T. gondii* in black goats was 16.76% (123/734, 95% CI: 14.06–19.46) with the titer ranged from 1:25 to 1:3200. The seroprevalence of *N. caninum* was 12.88% (76/590, 95% CI: 10.18–15.58). There was significant difference in seroprevalence of *N. caninum* in different regions (*P* < 0.01, χ^2^ = 30.63) and age groups (*P* < 0.05, χ^2^ = 11.85). Significant differences in seroprevalence of *T. gondii* were observed in different regions (*P* < 0.05, χ^2^ = 9.21) and different gender groups (*P* < 0.01, χ^2^ = 12.29). Results of seroprevalence of *T. gondii* and *N. caninum* indicated that *T. gondii* and *N. caninum* were prevalent parasites in black goats in Yunnan Province. This is the first report of seroprevalence of *T. gondii* and *N. caninum* in black goats in Yunnan Province. The results of this study indicated that some measures should be taken to control these two parasites and to reduce economic losses to the livestock industry in Yunnan Province.

## Introduction

*Toxoplasma gondii* and *Neospora caninum* are two obligate intracellular protozoan parasites infecting many animals. In addition, *T. gondii* is implicated in reproductive disorders in small ruminants, whereas *N. caninum* is considered an important pathogen causing abortion in dairy cows (1). Both parasites have a wide range of intermediate hosts including cattle, goats, sheep, other domestic and wild animals. Cats and dogs are the definitive hosts of *T. gondii* and *N. caninum*, respectively (1, 2). Animals can be infected by *T. gondii* through consumption of raw meat contained tissue cysts or ingestion of oocysts excreted by felines, and by vertical or transplacental transmission in intermediate hosts (3). In small ruminants, primary infection during pregnancy leads to serious congenital damage, resulting in abortion or stillbirth and negative economic impacts (4). A recent systematic review indicated that the global seroprevalence of *T. gondii* in goats was 27.49% (15,206/55,317, 95% CI: 24.15–30.95) (5). Despite goat is an important economic source of meat, fiber and milk in some countries worldwide, but a recent study indicated that there is still a higher potential to transmit *T. gondii* to humans by consumption of raw or undercooked meat, even small serving sizes (5 g) (6). To date, there was only one commercially available vaccine against *T. gondii* in sheep, but it has been discontinued due to self-limitation (3, 7). The average *T. gondii* seroprevalence in goats in China was 17.56% (3,260/18,556, 95% CI: 17.02–18.12) (8).

Similar to *T. gondii, N. caninum* has also been widely concerned and studied since it was first reported in 1984 (9). *N. caninum* can be transmitted horizontally and vertically in herds (10). *N. caninum* is considered as a major cause of abortion in cattle, particularly in dairy cattle, and studies revealed that 12% to 42% of aborted fetuses from dairy cattle were infected with *N. caninum* (1). Furthermore, goats would abort infectious fetuses when they were inoculated with *N. caninum* during pregnancy, and a meta-analysis revealed that the prevalence of *N. caninum* in aborted fetuses of goats was 7% worldwide (10, 11). Its zoonotic potential remains unknown because no evidence indicates that humans have been infected with *N. caninum* successfully (12, 13). The *N. caninum* seroprevalence in goats was estimated to be 5.99% (1,332/22,234, 95% CI: 4.38–7.83) worldwide (14). Because of *N. caninum* infection, the economic loss of beef and milk industries is approximate 1 billion US dollars annually (15).

Toxoplasmosis and neosporosis are cosmopolitan parasitic diseases and result in economic losses and reproductive reduction of the herds (1). China has the largest population of goats, and black goats are the most important economic goats in Yunnan Province, southwestern China. High density and diversity of domestic and wild animals might result in a high transmission risk of *T. gondii* and *N. caninum* in Yunnan Province. But knowledge on the seroprevalence of *T. gondii* and *N. caninum* in black goats in Yunnan Province is lacking. Therefore, the objectives of this study were to examine the seroprevalence of *T. gondii* and *N. caninum* and analyze the risk factors associated with their positivity in black goats in Yunnan Province.

## Materials and methods

### The investigation site and serum samples

**Figure 1:**
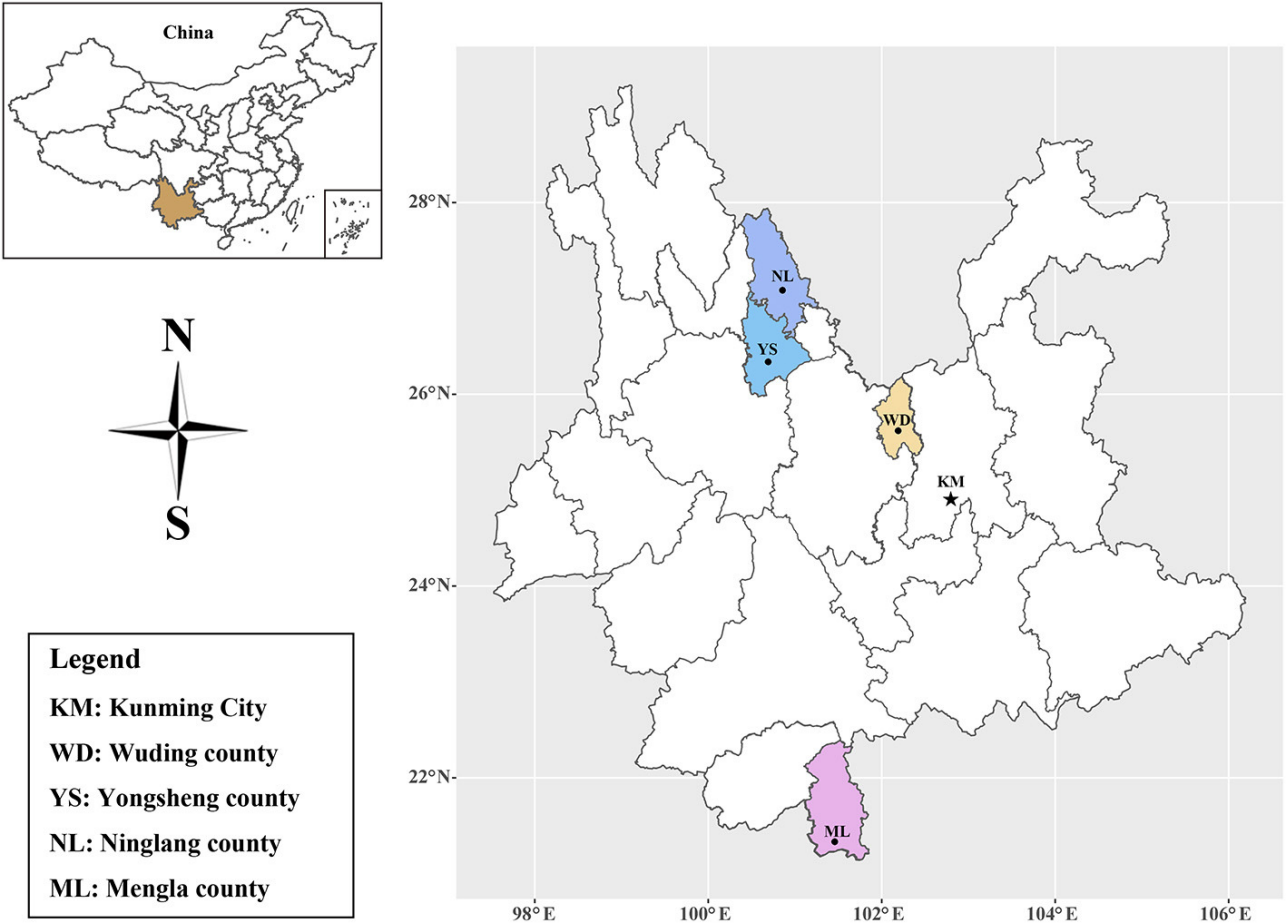
Distribution map of the sampling sites in Yunnan Province, Southwestern China.

Yunnan Province (97°31′ to 106°11′E, 21°8′ to 29°15′N), located in southwestern China, has a vast territory with diverse and unique natural resources. Yunnan Province has a subtropical monsoon climate, with an average annual temperature of 5 to 24°C and over 1000 mm of annual precipitation in most areas (http://www.yn.gov.cn/yngk/). After obtaining the permission of the farm owners or managers, 734 serum samples were collected from black goats in Wuding county (*n* = 479), Yongsheng county (*n* = 90), Ninglang county (*n* = 100) and Mengla county (*n* = 65) in Yunnan Province (Figure 1) from August to September, 2017. Approximately 5 mL of blood from each goat was sampled by jugular puncture in a tube without anticoagulant and stored at 4°C for 2 h, then centrifuged at 3,000 rpm for 10 min to collect serum samples, and all serum samples were stored at -20°C freezer until use.

### Serological examination

Modified agglutination test (MAT), the efficient method for diagnosis of toxoplasmosis (3), was used to detected the antibodies against *T. gondii* in this study. The antigen (formalin fixed tachyzoites) used in the experiment were kindly provided by Dr. Jitender P. Dubey (ARS, USDA). The MAT experiment was performed as described previously (16). Briefly, 2 μL serum sample was added to the first well of 96-well U bottomed reaction plate, then was diluted two-fold starting from 1:25 to 1:3,200. 25 μL antigen mixture was added to each well and the plates were incubated at 37°C for 12 h. The negative and positive control were contained in each plate. The serum with titer of 1:25 or higher was considered *T. gondii*-positive. The specific *N. caninum* antibodies were detected using an indirect ELISA kit (ID Screen^®^ *Neospora caninum* Indirect Multi-Species kit, ID VET, Montpellier, France) following the manufacturer's instructions. The kit has a high specificity and sensitivity (17). Positive and negative controls were set in each ELISA detection. The optical density (OD) was measured at 450 nm using microplate reader. The results were expressed as the ratio of absorbance of detected sample to the absorbance of the positive control following the formula: S/P = OD _sample_ – OD _negative control_ / OD _positive control_ – OD _negative control_. The samples with S/P % ≥50% were judged as positive.

### Statistical analysis

Chi-square (χ^2^) tests in SPSS software (release 23.0 standard version; SPSS, Inc., Chicago) were used for analyzing the variables (region, gender and age) associated with *T. gondii* and *N. caninum* infection. The variable with *P* < 0.05 was considered statistically significant. Results are presented as adjusted odds ratios (OR) with 95% confidence intervals (95% CI).

## Results

### The seroprevalence of *T. gondii* and *N. caninum* in black goats

In the present study, the overall seroprevalence of *T. gondii* and *N. caninum* in black goats was 16.76% (95% CI: 14.06–19.46) (Table 1) and 12.88% (95% CI: 10.18–15.58) (Table 2), respectively. Regarding the four study regions, the goats in Wuding county has the lowest seroprevalence of both *T. gondii* (13.78%, 66/479) and *N. caninum* (8.57%, 33/385), whereas the highest seroprevalence of *T. gondii* and *N. caninum* was detected in goats in Yongsheng county (24.44%, 22/90) and Ninglang county (33.33%, 18/54), respectively. Between gender groups, higher seroprevalence of *T. gondii* and *N. caninum* was detected in female goats, with 20.58% (93/452) and 14.80% (53/358), respectively. In addition, the highest seroprevalence of *T. gondii* was detected in goats aged more than 3 years (26.32%, 20/76); however, the seroprevalence of *N. caninum* in goats older than 3 years of age (14.71%, 10/68) was close to that of goats 1 to 2 years old (14.84%, 42/283), and both were significantly higher than that in goats of <1 year old (1.96%, 1/51).

**Table 1:** Seroprevalence of *Toxoplasma gondii* in black goats in Yunnan Province, Southwestern China.

**Factor**	**Category**	**Tested No**.	**Positive No**.	**Prevalence (%)**	**95% CI (%)**	**OR (95%)**	***P*** **-value**
Region	Wuding	479	66	13.78	10.69–16.87	Reference	0.027
	Yongsheng	90	22	24.44	15.56–33.32	2.03 (1.17–3.50)	
	Mengla	65	14	21.54	11.55–31.53	1.72 (0.90–3.28)	
	Ninglang	100	21	21.00	13.02–28.98	1.66 (0.96–2.87)	
Gender	Male	282	30	10.64	7.04–14.24	Reference	0.000
	Female	452	93	20.58	16.85–24.31	2.18 (1.40–3.39)	
Age	0<year<1	53	7	13.21	4.09–22.33	Reference	0.214
	1≤ year<2	356	57	16.01	12.20–19.82	1.25 (0.54–2.91)	
	2≤ year<3	230	36	15.65	10.95–20.35	1.22 (0.51–2.91)	
	year ≥3	76	20	26.32	16.42–36.22	2.35 (0.91–6.04)	
	year = unknown	19	3	15.79	0.61–32.19	1.23 (0.28–5.34)	
Total		734	123	16.76	14.06–19.46		

**Table 2:** Seroprevalence of *Neospora caninum* in black goats in Yunnan Province, Southwestern China.

**Factor**	**Category**	**Tested No**.	**Positive No**.	**Prevalence (%)**	**95% CI (%)**	**OR (95%)**	***P*** **-value**
Region	Wuding	385	33	8.57	5.77–11.37	Reference	0.000
	Yongsheng	91	12	13.19	6.24–20.14	1.62 (0.80–3.28)	
	Mengla	60	13	21.67	11.25–32.09	2.95 (1.45–6.00)	
	Ninglang	54	18	33.33	20.76–45.90	5.33 (2.73–10.41)	
Gender	Male	232	23	9.91	6.07–13.75	Reference	0.083
	Female	358	53	14.80	11.12–18.48	1.58 (0.94–2.66)	
Age	0<year<1	51	1	1.96	1.84–5.76	Reference	0.018
	1≤ year<2	283	42	14.84	10.70–18.98	8.71 (1.17–64.80)	
	2≤ year<3	184	21	11.41	6.82–16.00	6.44 (0.85–49.10)	
	year ≥3	68	10	14.71	6.29–23.13	8.62 (1.07–69.71)	
	year = unknown	4	2	50.00	1.00–99.00	50.0 (3.09–810.5)	
Total		590	76	12.88	10.18–15.58		

### Risk factors analysis

Statistical analysis showed that the seroprevalence of *T. gondii* in female goats was 20.58% (93/452), which was significantly higher than that in male goats (10.64%, 30/282) (χ^2^ = 12.29, df = 1, *P* < 0.001). There was statistically significant difference in seroprevalence of *T. gondii* among four study regions (χ^2^ = 9.21, df = 3, *P* < 0.05); but no statistically significant difference in *T. gondii* seroprevalence was observed between goats of different age groups (χ^2^ = 5.81, df = 1, *P* = 0.21) (Table 1). Moreover, statistically significant difference in seroprevalence of *N. caninum* was observed among different counties (χ^2^ = 3.64, df = 3, *P* < 0.001) and different age groups (χ^2^ = 11.85, df = 1, *P* < 0.05); whereas no statistically significant difference in *N. caninum* seroprevalence was found between the two genders (χ^2^ = 3.00, df = 1, *P* = 0.083).

## Discussion

Toxoplasmosis is a widely distributed zoonosis, both toxoplasmosis and neosporosis are two major causes of reproductive losses in small ruminants (18). In this study, we examined the seroprevalence of *T. gondii* and *N. caninum* in black goats in Yunnan Province, southwestern China, revealing the presence and relatively high seroprevalence of both parasites in study areas.

In the present study, the overall *T. gondii* seroprevalences in the examined black goats in Yunnan Province was 16.76%. A recent systematic review revealed that the seroprevalence of *T. gondii* in goats worldwide from 2000 to 2020 was 27.49%; of which, the highest and lowest seroprevalence of *T. gondii* in goats was detected in central America (62.15%) and Asia (20.74%), respectively (5). The seroprevalence of *T. gondii* in black goats in Yunnan Province detected in this study was higher than that in goats in Myanmar (11.39%, 32/281) (19), Korea (5.08%, 31/610) (20), Hunan Province (11.61%, 124/1,068) (21), Hubei Province (13.40%, 807/6,021) (22) and Shaanxi Province (14.11%, 106/751) (23) of China. But the *T. gondii* prevalence in black goats was lower than that in India (42.47%, 189/445) (24), Pakistan (42.83%, 227/530) (25), Mongolia (32.00%, 345/1,078) (26), Taiwan Province (32.22%, 203/630) (27) and Qinghai Province (29.54%, 192/650) of China (28). The seroprevalence of *T. gondii* detected in black goats in Yunnan Province in this study was similar to that detected in goats in Bangladesh (16.00%, 48/300) (29), and Yunnan Province (17.60%, 69/392) of China (30). The difference in *T. gondii* seroprevalence among difference regions may be related to different climate conditions, rearing conditions and breed variations.

The results of the present study demonstrated that there was significant difference in *T. gondii* seroprevalence of black goats from different geographical regions (*P* < 0.05) (Table 1). The difference may be caused by terrain and climate differences in Yunnan Province that has abundant rainfall and numerous lakes, and the annual average temperatures range from 5 to 24°C with humid climate. These environmental factors could be beneficial to the sporulation, viability and spread of *T. gondii* oocysts (31). Previous studies indicated that annual temperature and rainfall could facilitate the survival of the environmental *T. gondii* oocysts, and seasonally or permanently pasture goats as well as increases the contact between goats and the oocysts (32–34).

The majority of the black goats examined in this study were free-ranged, which could increase the risk of *T. gondii* infection *via* frequent contact with other free-ranged animals. The high seroprevalence of *T. gondii* in black goats in this study may due to the presence of cats in the farms (35, 36). Cats are the definitive host of *T. gondii*, and oocysts of *T. gondii* are shed *via* feces. The ruminants may be infected by ingesting oocysts (37).

In the present study, the highest *T. gondii* seroprevalence was found in black goats aged 3 years and more (26.32%), with seropositive rate 2.35 times higher than lambs aged lower than 1 year (95% CI = 0.91–6.04). This result is consistent with previous reports that younger goats had lower seropositivity than old goats (24, 36). In contrast, age as a significant risk factor of toxoplasmosis in older animals of ruminant species (i.e., cattle, sheep and goat) comparing to younger animals was observed in several studies (38, 39). Spišák et al. (40) found that the *T. gondii* prevalence in older goats (over 6 years of age) in Slovakia was 4.3 times higher than goats of up to 3 years age. Similar association between the *T. gondii* seroprevalence and age has also been observed in cattle, sheep, goats and pigs in Portugal (41). Furthermore, a recent meta-analysis demonstrated that goats older than 1 year of age were at higher risk of being infected with *T. gondii*, because long-term exposure to the pasture increases the opportunity of ingesting oocysts (5). The results of the present study indicated that *T. gondii* infection was common in black goats in Yunnan Province (Table 1).

A recent systematic review reported that higher *T. gondii* prevalence was observed in female goats than that in males (OR = 1.43; 95% CI = 1.23–1.65) (5). In this study, the seroprevalence of *T. gondii* in female goats was 2.18 times higher than that in male goats (Table 2) (95% CI = 1.40–3.39; *P* < 0.01). This result was consistent with previous studies in sheep and goats in which male animals had a lower *T. gondii* prevalence than the females (42, 43). Some studies inferred that higher seroprevalence in females might be associated with their longer life for milk production and reproduction, whereas males are slaughtered for meat supply at an earlier age (24, 44). In addition, a previous study indicated that hormone differences may increase their susceptibility to *T. gondii* (45).

In this study, the overall seroprevalence of *N. caninum* in black goats was 12.88% (Table 2), which was higher than that in goats in Poland (9.00%, 95/1,060) (46), Pakistan (9.15%, 13/142) (47), Argentina (6.65%, 106/1,594) (48), south America (6.35%, 25/394) (49), Spain (6.00%, 3/50) (32), Brazil (4.58%, 30/655) (50), Turkey (3.21%, 8/249) (51), Romania (2.34%, 12/512) (52), Greece (6.93%, 26/375) (18) and Jordan (1.99%, 6/302) (53). However, it was lower than that in the Czech Republic (18.57%, 13/70) (35). Growing evidences indicated that the risk of infection for *N. caninum* is linked to the age of the hosts, rearing system, worming, the time of exposure to the parasite and the contact with dogs around the farms and history of abortion (18, 54). Also, goat breeds, climatic conditions, feeding and management conditions might contribute to the different seroprevalence of *N. caninum* in black goats in Yunnan Province, and more studies are warranted to investigate the potential association. In addition, previous studies have reported that many birds (e.g., domestic chickens and many wild birds) can act as intermediate hosts of *N. caninum* and can transmit the pathogen after being preyed upon by dogs when they are foraging on the ground, thus facilitating the spread of the *N. caninum* (55–57). However, the potential role played by birds in the transmission of *N. caninum* in Yunnan Province needs further study in the future.

In this study, the region and age factors were significantly related to *N. caninum* infection in black goats (*P* < 0.05). The seroprevalence of *N. caninum* in black goats aged 0–1 years (1.96%) was significantly lower than those in 1–2 years group (14.84%) and 2–3 years group (11.41%). These results were consistent with a previous report (41). Regarding the age groups, adult black goats showed the higher *N. caninum* seroprevalence than lambs, which is consistent with the results that adult cattle and heifers/steers have higher infection rate than calves, due to the increasing chances in postnatal oocyst infection with age (58). Similar to our results, the lowest *N. caninum* seroprevalence was also found in yaks of the 0–1 year group (59). However, two previous reports indicated that no statistically significant difference was observed in *N. caninum* seroprevalence between cattle of different age groups, but the *N. caninum* seroprevalence was strongly linked with the factors of abortion, parity number, gestation number and number of lactations (60, 61). Thus, a comprehensive study should be performed in the future to elucidate the important role of age in *N. caninum* epidemiology. With respect to regions, the present study found that there was significant difference in *N. caninum* seroprevalence among different study areas (*P* < 0.001). We speculated that rearing system, management measures, presence of dogs, and even the history of abortion in goats in the study areas may contribute to the difference in seroprevalence. Nevertheless, the region factor is a complex of multi-subfactors, including climatic, environmental factors. In Italy, the climatic and environmental factors were determined to influence the *N. caninum* distribution in cattle by geographical information system (GIS) and remote sensing (RS) technology (58). Additionally, Villa et al. (62) found a correlation between the geographic distance of the sampling sites and genetic distance of *N. caninum*, further explaining the possible reasons for the seroprevalence difference among different regions. In our investigation, no statistically significant difference in *N. caninum* seroprevalence was observed between black goats of different genders (*P* = 0.083). Similar to our findings, Shireen et al. (63) indicated that there was no significant correlation between *N. caninum* and gender in small ruminants in Egypt.

In the present study, the co-infection rate of *T. gondii* and *N. caninum* in black goats was 4.44% (26/585), which was similar to the across-infection rate of *T. gondii* and *N. caninum* previous detected in black-bone sheep and goats in Yunnan Province (3.63%, 17/468) (64). Also, it was slightly lower than that in Qinghai Province, where the co-infection rate of *T. gondii* and *N. caninum* was 5.23% in goats and 6.5% in sheep (28). Sampling sizes, grazing practices, the presence of dogs and cats may be the important factors that contribute to co-infection of *N. caninum* and *T. gondii* (1, 65). However, based on an *in vitro*, immunological and serological experimental study, researches indicated that there is no exclusivity of infection and co-infection is a random event (66).

## Conclusion

The present study examined the seroprevalence of *T. gondii* and *N. caninum* infection in black goats in Yunnan Province by using MAT and indirect ELISA methods. The overall seroprevalences of *T. gondii* and *N. caninum* in black goats were 16.76 and 12.88%, respectively. Region and gender were significantly associated with *T. gondii* infection in black goats, while region and age were significantly associated with *N. caninum* seroprevalence in black goats. The results of the present study demonstrated that *T. gondii* and *N. caninum* were highly prevalent in black goats in Yunnan Province. Therefore, integrated measures should be taken to prevent and control infection of black goats with these two parasites.

## Data availability statement

The original contributions presented in the study are included in the article/supplementary material, further inquiries can be directed to the corresponding authors.

## Ethics statement

The animal study was reviewed and approved by Animal Ethics and Welfare Committee of Yunnan Agricultural University.

## Author contributions

J-JH, F-CZ, and X-QZ designed the study and revised the manuscript. X-HH and S-CX performed the experiments, analyzed the data, and wrote the manuscript. Q-LL, L-XS, ZL, and J-FY participated in implementation of the study. All authors read and approved the final version of the manuscript.

## Funding

Project support was provided by the Yunnan Expert Workstation (Grant No. 202005AF150041), the Veterinary Public Health Innovation Team of Yunnan Province (Grant No. 202105AE160014), the Fund for Shanxi 1331 Project (Grant No. 20211331-13) and the Agricultural Science and Technology Innovation Program (ASTIP) (Grant No. CAAS-ASTIP-2016-LVRI-03). The funders had no role in the design of the study; in the collection, analyses, or interpretation of data; in the writing of the manuscript, or in the decision to publish the results.

## Conflict of interest

The authors declare that the research was conducted in the absence of any commercial or financial relationships that could be construed as a potential conflict of interest.

## Publisher's note

All claims expressed in this article are solely those of the authors and do not necessarily represent those of their affiliated organizations, or those of the publisher, the editors and the reviewers. Any product that may be evaluated in this article, or claim that may be made by its manufacturer, is not guaranteed or endorsed by the publisher.
